# Rapamycin increases survival in ALS mice lacking mature lymphocytes

**DOI:** 10.1186/1750-1326-8-31

**Published:** 2013-09-11

**Authors:** Kim A Staats, Sara Hernandez, Susann Schönefeldt, André Bento-Abreu, James Dooley, Philip Van Damme, Adrian Liston, Wim Robberecht, Ludo Van Den Bosch

**Affiliations:** 1 Laboratory of Neurobiology, Vesalius Research Center, VIB, Leuven, Belgium; 2 Experimental Neurology (Department of Neurosciences), Leuven Research Institute for Neuroscience and Disease (LIND), University of Leuven (KU Leuven), Leuven, Belgium; 3 Autoimmune Genetics Laboratory, VIB, Leuven, Belgium; 4 Department of Microbiology and Immunology, University of Leuven (KU Leuven), Leuven, Belgium; 5 Department of Neurology, University Hospitals Leuven, Leuven, Belgium

**Keywords:** Autophagy, Rapamycin, Neurodegeneration, Amyotrophic lateral sclerosis, Motor neuron disease, Sirolimus, Rapamune, Immunosuppression

## Abstract

**Background:**

Amyotrophic Lateral Sclerosis (ALS) is a devastating progressive neurodegenerative disease. Disease pathophysiology is complex and not yet fully understood, but is proposed to include the accumulation of misfolded proteins, as aggregates are present in spinal cords from ALS patients and in ALS model organisms. Increasing autophagy is hypothesized to be protective in ALS as it removes these aggregates. Rapamycin is frequently used to increase autophagy, but is also a potent immune suppressor. To properly assess the role of rapamycin-induced autophagy, the immune suppressive role of rapamycin should be negated.

**Findings:**

Autophagy is increased in the spinal cord of ALS mice. Dietary supplementation of rapamycin increases autophagy, but does not increase the survival of mutant SOD1 mice. To measure the effect of rapamycin in ALS independent of immunosuppression, we tested the effect of rapamycin in ALS mice deficient of mature lymphocytes. Our results show that rapamycin moderately increases the survival of these ALS mice deficient of mature lymphocytes.

**Conclusions:**

Rapamycin could suppress protective immune responses while enhancing protective autophagy reactions during the ALS disease process. While these opposing effects can cancel each other out, the use of immunodeficient mice allows segregation of effects. Our results indicate that maximal therapeutic benefit may be achieved through the use of compounds that enhance autophagy without causing immune suppression.

## Findings

Amyotrophic Lateral Sclerosis (ALS) is a devastating progressive neurodegenerative disease, which primarily involves the loss of motor neurons and denervation of muscle fibers, resulting in muscle weakness and paralysis. The disease has an annual incidence of 2.7 cases per 100,000 people in Europe [1] and most patients succumb to the disease within 3 to 5 years after onset. On average 10% of all ALS cases are familial, of which 20% are caused by mutations in the superoxide dismutase 1 (SOD1) gene. Based on these mutations, ALS rodent models have been generated that predictably mimic the patient disease process [2]. As disease progression is indistinguishable between familial and sporadic cases, common disease mechanisms are expected. Two of these mechanisms are aggregation [3] and the impaired clearance of misfolded proteins [4].

A process to induce clearance of aggregated or misfolded proteins is macroautophagy (further described as autophagy). This is an intracellular clearance mechanism to degrade long-lived proteins and organelles. Autophagy is increased in cells expressing (mutant) ALS genes *in vitro*[5], in the spinal cord of ALS mice [6-8] and of ALS patients [9]. Increasing autophagy is beneficial in neurodegenerative disease models, including those for Alzheimer’s disease [10], Parkinson’s disease [10], spinal cerebellar ataxia 3 [11], Huntington’s disease [12] and frontotemporal lobar dementia [13]. Also in ALS mice, genetically increasing autophagy in neurons increases survival [14]. Pharmacologically, increasing autophagy in ALS mice has not yet provided similar beneficial results.

Rapamycin is frequently used to pharmacologically increase autophagy by inhibiting the phosphorylation of the mammalian target of rapamycin (mTOR) [15]. In ALS mice, this drug has severely decreased survival [6] or did not affect survival [16]. Rapamycin is additionally used as a potent immunosuppressant as it inhibits the activation of T-cells [17]. Interestingly, removal of mature lymphocytes or functional T-cells in ALS mice decreases survival [18,19] and thus rapamycin may be, in part, detrimental in ALS due to its immunosuppressive action. Dietary restriction experiments on ALS mice have shown to increase autophagy and decrease ALS mouse survival [20]. These studies may also be influenced by immunosuppression as dietary restriction also decreases activation of mTOR [21].

**Figure 1:**
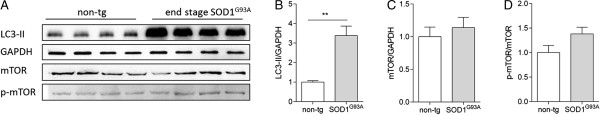
**Autophagy is increased in ALS mouse spinal cord.** Western blot analysis of age-matched spinal cords of non-transgenic (non-tg, n = 4) and end stage SOD1^G93A^ mice (n = 4) **(A)**. Quantification of Western blot signal of LC3-II for non-transgenic and end stage SOD1^G93A^ mice **(B)**, mTOR **(C)** and p-mTOR **(D)**. **p < 0.01.

To confirm whether autophagy is increased in SOD1^G93A^ mice, we performed Western blot analysis. The lipid-bound form of microtubule-associated proteins 1A/1B light chain (LC3-II) is increased in the spinal cord of end stage SOD1^G93A^ compared to age-matched non-transgenic mice (Figure 1A & B). mTOR is similarly expressed at end stage (Figure 1A & C) as is the phosphorylation of this receptor (Figure 1A & D).

**Figure 2:**
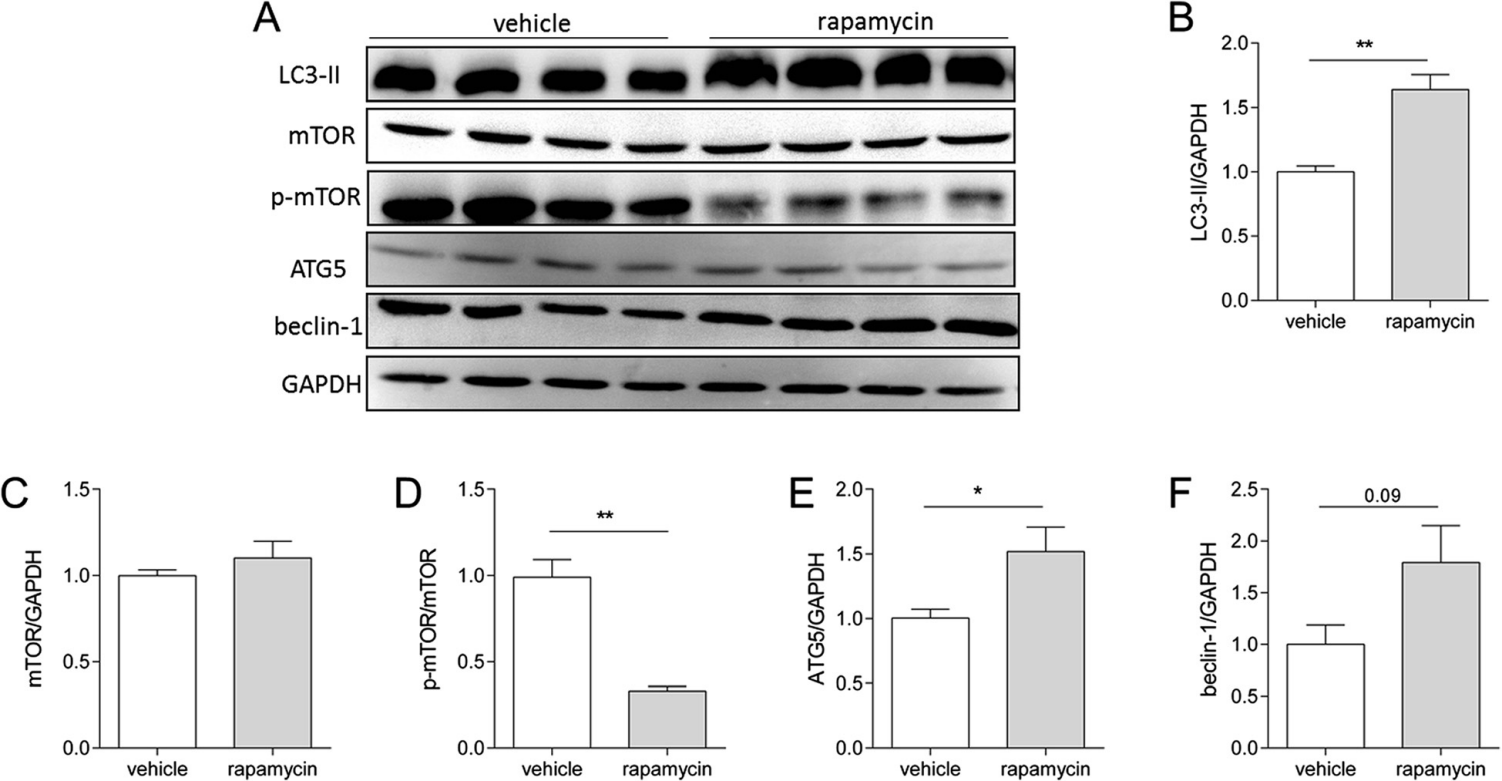
**Rapamycin delivered in chow increases autophagy in the spinal cord of RAG1**^**−/− **^**mice.** Western blot analysis of LC3-II, mTOR and p-mTOR in the spinal cords of RAG1^−/−^ mice fed vehicle or rapamycin containing chow for 3 months **(A)**. Quantification of the levels of LC3-II in spinal cords of mice fed chow containing rapamycin for 3 months (n = 4) or vehicle chow (n = 4) **(B)**. Quantification of mTOR **(C)**, p-mTOR **(D)**, ATG5 **(E)** and beclin-1 **(F)**. *p < 0.05, **p < 0.01.

Next, we confirmed that dietary supplementation of rapamycin increases autophagy. Increased levels of LC3-II are detected in spinal cords of RAG1^−/−^ mice treated with rapamycin (Figure 2B). The expression of mTOR remained constant (Figure 2C), although the phosphorylation of the receptor was decreased by rapamycin (Figure 2D). Additional markers of autophagy, ATG5 and beclin-1, are also increased by rapamycin (Figure 2E & F).

**Figure 3:**
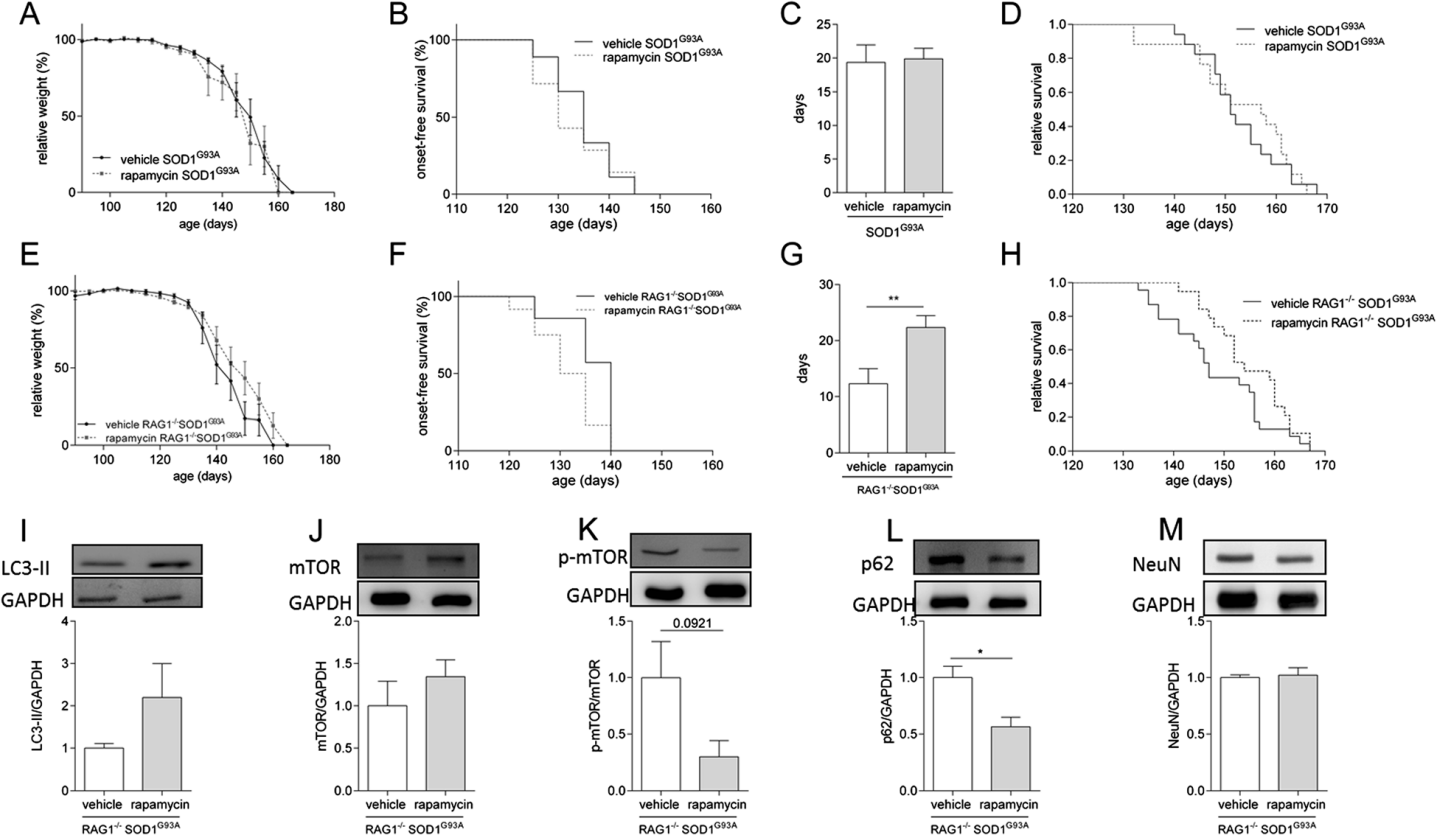
**Rapamycin does not affect survival of SOD1**^**G93A **^**mice, but increases survival of SOD1**^**G93A **^**mice lacking mature lymphocytes.** SOD1^G93A^ mice fed vehicle (n = 9) or rapamycin chow (n = 7) relative weight **(A)**, onset-free survival **(B)** and disease duration **(C)**. RAG1^−/−^ SOD1^G93A^ mice fed vehicle (n = 7) or rapamycin chow (n = 12) relative weight **(E)**, onset-free survival **(F)** and disease duration **(G)**. Survival analysis of SOD1^G93A^ mice that were fed vehicle (152.6 ± 1.8 days, n = 17) or rapamycin chow (153.1 ± 2.5 days, n = 17) **(D)**. Survival analysis of RAG^−/−^ SOD1^G93A^ mice fed with vehicle (148.6 ± 2.0 days, n = 23) or rapamycin chow (155.1 ± 1.8 days, n = 19, p = 0.04) **(H)**. Western blot analysis of the levels of LC3-II **(I)**, mTOR **(J)**, phosphorylated mTOR (p-mTOR) **(K)**, p62 **(L)** and NeuN **(M)** for end stage RAG1^−/−^ SOD1^G93A^ mice fed vehicle (n = 4) or rapamycin-containing chow (n = 4 and n = 5 for the analysis of p62 and NeuN). *p < 0.05, **p < 0.01.

To assess the effect of increased autophagy in ALS, we treated pre-symptomatic SOD1^G93A^ mice with rapamycin. Rapamycin does not affect disease onset (Figure 3B), disease duration (Figure 3C) or survival of SOD1^G93A^ mice compared to SOD1^G93A^ mice fed vehicle diet (Figure 3D). However, a potential protective effect of increased autophagy by rapamycin in SOD1^G93A^ mice may be masked by the detrimental immunosuppressive effect of rapamycin on lymphocytes in SOD1^G93A^ mice. To circumvent this effect of rapamycin, we crossbred RAG1^−/−^ mice, which are devoid of mature lymphocytes [22], with SOD1^G93A^ mice to assess the effect of rapamycin in the absence of mature lymphocytes. Interestingly, when the immunosuppressive effect of rapamycin on lymphocytes cannot be exerted (as is the case in RAG1^−/−^ mice), rapamycin significantly prolongs disease duration (Figure 3G) and survival with 6.5 days (Figure 3H), while it does not affect disease onset (Figure 3F). Despite that ALS mice may consume less chow as they approach end stage, a trend is shown for increased autophagy in the spinal cords of RAG1^−/−^ SOD1^G93A^ mice on rapamycin-containing chow (Figure 3I-L). Additionally, RAG1^−/−^ SOD1^G93A^ mice fed rapamycin-containing chow have a similar amount of neurons in the spinal cord at end stage (Figure 3M), suggesting these mice did not become end stage due to other reasons than neuronal loss.

The slight increase of survival of RAG1^−/−^ SOD1^G93A^ mice fed rapamycin-containing chow implies that the beneficial effect of increasing autophagy in SOD1^G93A^ mice may be masked by the immunosuppressive effect of rapamycin in mice with mature lymphocytes. In line with this hypothesis, a recent study that assessed the effect of rapamycin on ALS mice showed a decreased survival of more than 2 weeks [6]. This is comparable to the size of the effect on survival detected by others after removing mature lymphocytes from ALS mice [23]. In our mice, the survival of vehicle treated SOD1^G93A^ and RAG1^−/−^ SOD1^G93A^ mice do not significantly differ (p = 0.20), although there is a trend that RAG1^−/−^ SOD1^G93A^ mice live slightly shorter (4.0 days).

The dual effect of rapamycin (immunosuppression and increased autophagy) is a contraindication to use this drug for ALS patients and thus the development of compounds that specifically target autophagy without immunosuppression is essential. As removing T-cells may be detrimental in ALS, RAG1^−/−^ mice are useful to assess the role of autophagy in different disease models, such as in inclusion body myopathy [24], until specific autophagy-inducing compounds become available.

In summary, a protective effect of increasing autophagy is expected in ALS, but not yet been demonstrated pharmacologically *in vivo*. We circumvented the negative effect of rapamycin on lymphocytes by removing these cells from SOD1^G93A^ mice and found a moderate but significant effect on survival. This protective effect seems to be due to increased autophagy and indicates that this could become a therapeutic strategy to treat ALS.

## Methods

### Animal testing

Mice overexpressing SOD1^G93A^ and recombination activating gene 1 knockout (RAG1^−/−^) mice were purchased from Jackson Laboratories (Bar Harbor, USA) and maintained on a C57BL/6 background. Chow and water were provided *ad libitum* and mice were housed in the specific pathogen free facility of KU Leuven. A decrease of 10% in body weight compared to their average between day 90 and 105 is considered as disease onset. Mice no longer surviving were assessed as 0 g. End stage is defined as the age when mice could no longer right themselves from their back within 10 s and this is the measurement of survival. For Figure 3A-E both RAG1^+/−^ SOD1^G93A^ and RAG1^+/+^ SOD1^G93A^ mice were used, as their survival does not differ. All experiments were performed with the approval of the Animal Ethical Committee of KU Leuven (P020/2010).

### Diet preparation

Rapamycin (LC Labs) was encapsulated by Southwest Research Institute (San Antonio, USA) with coating material Eudragit S100 (Röhm Pharma) as described previously [25]. Encapsulated rapamycin was processed in Purina 5LG6 mouse chow by TestDiet (London, UK) at a concentration of 14 mg/kg food (2.33 mg of rapamycin per kg body weight per day, assuming a body weight of 30 g and a daily consumption of 5 g per mouse). Rapamycin or vehicle chow substituted the animal house chow *ad libitum* from 60 days of age until end stage or 160 days.

### Western blot

Samples were size-separated through denaturing sodium dodecyl sulfate polyacrylamide gel electrophoresis. Protein was electro-transferred to a nitrocellulose membrane in Tris–glycine–methanol buffer and processed with the Supersignal ChemiLuminiscence detection kit (Pierce Biotechnology Inc.). The following antibodies were used: anti LC3-II, anti mTOR, anti p-mTOR, anti Beclin, anti ATG5 and anti p-62 (Cell Signalling).

### Statistical analysis

The statistical analysis was performed with Graphpad Prism (version 5.04) software. Unpaired 2-sided Student’s t-tests were used to analyse differences between 2 groups and the Gehan-Breslow-Wilcoxon for survival data. Significance is assumed for p ≤ 0.05. Values are shown as mean ± standard error of the mean.

## Abbreviations

ALS: Amyotrophic lateral sclerosis; ATG5: Autophagy protein 5; LC3: Microtubule-associated proteins 1A/1B light chain; mTOR: Mammalian target of rapamycin; p-mTOR: Phosphorylated form of the mammalian target of rapamycin; NeuN: Neuronal nuclear antigen (Feminizing Locus on X-3); SOD1: Superoxide dismutase 1.

## Competing interests

The authors declare that they have no competing interests.

## Authors’ contributions

KAS, SS and JD performed the murine behavioural experiments. SH and ABA performed the Western blot analysis. KAS, PVD, WR, AL and LVDB participated in the design of the study. KAS and LVDB participated in preparation of the manuscript. All authors read and approved the final manuscript.
